# Supplementary material for: Co-delivery of D-(KLAKLAK)2 Peptide and Chlorin e6 using a Liposomal Complex for Synergistic Cancer Therapy
Source: Pharmaceutics. 2019 Jun 21;11(6):293. doi: 10.3390/pharmaceutics11060293 (PMC6630662; doi:10.3390/pharmaceutics11060293)
Supplement: Supplementary file 1 [file pharmaceutics-11-00293-s001.pdf]

pharmaceutics

Supplementary Materials: Co-delivery of D-(KLAKLAK)$_2$ Peptide and Chlorin e6 using a Liposomal Complex for Synergistic Cancer Therapy

Chaemin Lim, Jin Kook Kang, Woong Roeck Won, June Yong Park, Sang Myung Han, Thi ngoc Le, Jae Chang Kim, Jaewon Her, Yuseon Shin and Kyung Taek Oh

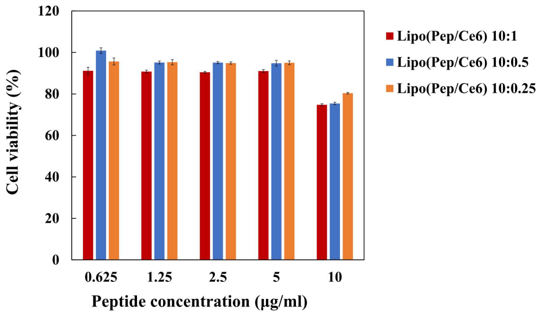

Figure S1. The cell viability of KB cells treated with Lipo (Pep, Ce6) at different drug loading ratio.
